# Editorial: Artificial Intelligence Bioinformatics: Development and Application of Tools for Omics and Inter-Omics Studies

**DOI:** 10.3389/fgene.2020.00309

**Published:** 2020-04-09

**Authors:** Davide Chicco, Dominik Heider, Angelo Facchiano

**Affiliations:** ^1^Krembil Research Institute, University Health Network, Toronto, ON, Canada; ^2^Department of Mathematics and Computer Science, Philipps-University of Marburg, Marburg, Germany; ^3^Istituto di Scienze dell'Alimentazione, Consiglio Nazionale delle Ricerche (CNR), Avellino, Italy

**Keywords:** artificial intelligence, bioinformatics, genomics, omics, inter-omics, machine learning, data mining, proteomics

For half a century, bioinformatics and computational biology have provided tools and data analysis approaches, so the beginning of the omics era represented a novel challenge for researchers, that converged to the area of bioinformatics from the fields of informatics, mathematics, and statistics. In most cases, the solutions offered appeared difficult to use for researchers working in biomedical areas. This occurred in particular when sophisticated approaches from the field of data science and artificial intelligence (AI), were applied to biomedical data (Lisboa et al., 2000).

Machine learning, statistical learning, and soft-computing approaches, such as deep neural networks or genetic algorithms, have also become terms used in the *bio* world, with an incomplete comprehension however, of their potential (Pavel et al., 2016; Lin and Lane, 2017; Zeng and Lumley, 2018). In recent years, omics, multi-omics, and inter-omics experiments have presented a further step toward the investigation in biology, opening the window on personalized medicine, for example for diagnostics (Riemenschneider et al., 2016). The era of big data in medicine is imminent and represents yet a further step forward. Considering this, our Research Topic presents articles on novel developments in the field of artificial intelligence in biology and medicine, and their applications in the analysis of high-throughput data from omics and inter-omics approaches (Facchiano et al.).

## 1. The Article Collection

The Research Topic includes 13 articles:

- 7 Original Research articles (Di Filippo et al.; Kong et al.; Leclercq et al.; Liu et al.; Maj et al.; Simidjievski et al.; Xu et al.)
- 1 Brief Research Report article (Quinn et al.)
- 1 Methods article (Niu et al.)
- 2 Technology and Code articles (Martin and Heider; Wang et al.)
- 1 Review article (M'sch et al.)
- 1 Systematic Review article (Zeng and Bromberg).

The published articles have been evaluated according to each journal editorial policy, by experts of the field. The Research Topic received seven other manuscripts, judged unsuitable for publication and rejected during the review process. The submission deadline was 29th June 2019, therefore any data, experiment, and result presented in the Research Topic articles must be in reference to data, experiments, and results obtained earlier than that date.

### 1.1. Original Scientific Research and Methods

Simidjievski et al. showed how variational autoencoders (VAEs) can be employed to integrate heterogeneous cancer data. They used these artificial neural networks to integrate multi-omics data such as somatic copy number aberrations (CNA), messenger RNA (mRNA) expressions, and clinical data of patients diagnosed with breast cancer from the METABRIC initiative (Curtis et al., 2012).

Di Filippo et al. developed an R shiny app named HiCeekR that can be used for the analyses of Hi-C data. In contrast to existing tools, HiCeekR represents an easy-to-use graphical user interface to a complete Hi-C data analysis pipeline, including all relevant analysis and visualization steps.

In their article, Niu et al. developed and analyzed a novel pre-training-retraining strategy for deep neural networks and evaluated this strategy based on the prediction of tissue-specific activation of cis-regulatory elements (CREs). This is a very important step as the number of tissue-specific samples is limited. They used all CREs for the pre-training of the net and then used transfer learning to improve tissue-specific predictions.

Maj et al. combined supervised and unsupervised machine learning models on tissue-specific cis-eQTL gene expression data to distinguish mild cognitive impairment and patients with Alzheimer's Disease and to detect potential biological associations.

Kong et al. developed a novel computational model for the prediction of protein-protein interactions (PPIs). The new method, FCTP-WSRC, used a combination of F-vector, composition (C), and transition (T) to numerically encode the protein sequences and subsequently uses principal component analysis (PCA) to extract features. The PCA representation is then used as an input for weighted sparse representation-based classification. FCTP-WSRC has been evaluated on several data sets and shows a superior prediction performance in terms of accuracy and computing time.

Liu et al. used multi-omics data, namely DNA methylation, copy number variation, and gene expression to identify dysfunctional subpathways in cancer and validated their findings with several cancer datasets, for example, liver hepatocellular carcinoma (LIHC), head-neck squamous cell carcinoma (HNSC), cervical squamous cell carcinoma, and endocervical adenocarcinoma.

Xu et al. identified dysregulated competitive endogenous RNA (ceRNA) interactions driven by copy number variation (CNV) in gliomas, and then found their associations with prognosis and histological subtypes by gene set enrichment analysis. Biological functions related to the oncogenesis of malignant gliomas have been detected by the functional analysis of the CNV-driven ceRNA network.

Leclercq et al. proposed BioDiscML, a software program that implements a machine learning method for discovery of biomarkers from multi-omics data. The automatic pipeline built up for mining signatures of diseases by classification, together with the feature selection processes for biomarker discovery, represent the main strengths of this work.

Quinn et al. described an anomaly detector for tissue transcriptomes, aimed to identify cancer without ever seeing a single cancer example. The outlier detection algorithm has been trained on normal samples from a large public data set (Lonsdale et al., 2013) and applied to classify cancer samples from another large public data set (Weinstein et al., 2013).

### 1.2. Technology Applications

Martin and Heider developed the ContraDRG software, available on a web server, that computationally emulates complex predictions in a reverse-engineering like manner, with intensive calculations using machine learning techniques. ContraDRG can be used to predict partial charges for small molecules based on molecular topology predictions from two commonly used tools, such as PRODRG and ATB. ContraDRG can accurately predict partial charges quickly, and thus can also be applied for screening projects with large amounts of molecules.

Wang et al. used convolutional neural networks to measure conditional relatedness, that is, the degree of the relation of a pair of genes in certain conditions and showed that this approach has a lower false-positive rate compared to traditional co-expression analyses, due to the combination of prior knowledge and co-expression.

### 1.3. Reviews

In their overview, M'sch et al. reported and described several applications of machine learning methods in immunotherapy, with special attention given to T cell receptor-mediated therapies. They list more than 150 references, which show several data sources and multiple computational intelligence algorithms employed for several goals such as proteasomal cleavage prediction, epitope prediction, and T-cell receptor prediction.

Zeng and Bromberg summarized the recent findings of the functional effects of synonymous mutations in genomes. In particular, they recapped the details and evaluated the performance of nine existing computational methods capable of predicting functional effects for synonymous mutations, also demonstrating the limitations of currently available tools.

## 2. Discussion

The Research Topic stands out because of its heterogeneity and the diversity of its contents: article authors applied different computational intelligence methods, on different datasets (almost all differing from source and type), to investigate different scientific bioinformatics questions. This diversity confirms the versatility of data mining usage and the huge number of biological subjects that need to be investigated and analyzed.

The Research Topic, in fact, includes original research articles applying statistical learning methods to several dataset types, with gene expression being the most frequent one (Liu et al.; Maj et al.; Quinn et al.; Simidjievski et al.; Wang et al.).

Some authors employed traditional biostatistics techniques, while others took advantage of machine learning methods. In particular, we report the frequent usage of deep learning and artificial neural networks among the applications described in the Research Topic (Leclercq et al.; Maj et al.; Niu et al.; Simidjievski et al.).

The Research Topic articles differ in data and software availability, too. The authors of three articles made their data and software openly public (Maj et al.; Niu et al.; Wang et al.). Two articles have only made their software publicly accessible, but not the data (Leclercq et al.; Simidjievski et al.). The authors of five articles made their datasets available to the scientific community, but not their software (Di Filippo et al.; Kong et al.; Martin and Heider; Quinn et al.; Xu et al.).

## Author Contributions

All authors listed have made a substantial, direct and intellectual contribution to the work, and approved it for publication.

### Conflict of Interest

The authors declare that the research was conducted in the absence of any commercial or financial relationships that could be construed as a potential conflict of interest.
